# Insanity in Child-Bearing

**Published:** 1887-05-07

**Authors:** Henry Rayner

**Affiliations:** Medical Superintendent, Hanwell Asylum


### INSANITY IN CHILD-BEARING.

By Henry Rayner, M.D., Medical Superintendent, Hanwell Asylum.

A Lecture delivered at the Midwives' Institute and Trained Nurses' Club.

### Part I.—Puerperal Forms.

Mental Disorders Classified.

The mental disorders associated with child-bearing might be divided or grouped into those occurring during gestation, delivery, child-bed, and nursing; or, to use more technical terms, into gestational, parturient, puerperal, and lactational insanities. He named these groups in the order that physiological sequence would give to them; but, for their purpose that evening, he proposed to give precedence to the puerperal forms, they being not only by far the most numerous, but having, he thought, much greater special interest for his hearers.

The Puerperal State.

The first question that suggested itself in regard to these insanities was, whether the puerperal state acted as a distinct and specific cause, producing a special variety of insanity, which could be recognised as differing from all other forms? Some writers held this to be the case, but others, more numerous, of greater authority, and of more recent date, denied that there was any such special form. In considering this question, they found that the forms of insanity occurring in the puerperal state were varied; that they could not be distinguished from similar forms resulting from other causes; and that ordinary causes played an active part in their production. Puerperal insanities, in fact, only possessed a family likeness, from occurring under similar conditions of nutritional and nervous exhaustion, and did not constitute a special disease. We used the term puerperal insanity, merely to indicate those insanities which occurred within six or eight weeks after confinement. The due appreciation of the value of a subject was the earliest point that should be fixed in our minds in approaching it. This was specially the case in medicine, so that he desired his hearers to have a definite estimate of the frequency of these disorders, that they might not fall into the error, often imputed to specialists (perhaps not utterly without reason), of seeing or anticipating the disease in which they were interested in every patient coming under observation, a state of things which would be as disagreeable to nurses as to those whom they might have under their charge.

Causes of the Puerperal State.

From the statistics of the Commissioners in Lunacy it appeared that the puerperal state was assigned as a cause in 6.5 per cent. of all cases admitted to asylums in England. The estimate had been made that one in every four hundred labours was followed by mental disorder,—a certainly excessive estimate. There could be no doubt that the mental disorder was more frequent in towns than among the country population. It was certainly less frequent in lying-in institutions than in the population at large. This might be due to the very favourable conditions,—the quiet, good nursing, absence from home anxieties, the regulated diet, etc. Then, again, it was said that it was more frequent in some countries than in others. France produced more puerperal insanity than England, and some counties of England yielded a larger proportion of insanity than others. Class or rank in life exercised an important influence, most observers agreeing that more cases occurred among the wealthy than among the poor, notwithstanding the great advantages, especially during child-bed, which the former possessed. Yet these advantages were more than counterbalanced by the ill-influence of luxurious living. Of the admissions to English asylums 7.4 per cent. of the private patients were ascribed to this cause, while only 6.4 per cent. of pauper cases thus originated. The difference was even greater than these figures showed, since many patients in the richer classes were treated out of asylums, and so did not appear in the statistics.

Hereditary Predisposition.

Hereditary influence played a very important part in the development of these insanities. Burrows estimated that 50 per cent. of the cases were due to hereditary influence, and the lowest estimate ascribed 30 per cent. to this cause. We found that, in insanity, special forms were specially inherited.

Illegitimacy.

An undue proportion of cases occurred in illegitimacy, a fact due probably to anxiety and mental distress. It was not uncommon to find a history of mental distress or anxiety from other causes during gestation in most cases which culminated in insanity during child-bed.

Recurrences of Insanity.

Nearly half the cases followed the first confinement, and a considerable proportion followed the second. There was, in fact, a tendency for the malady to recur with successive births. In one case he knew it occurred eight times; in another six times. When it recurred three or four times, the recoveries were generally incomplete, and such cases ended in chronic insanity. Usually, however, the second attack was lighter than the first, and the danger then diminished with each succeeding child. Predisposition to insanity was increased by age at the first delivery. The nearer to forty, the greater was the danger. In women, however, who had borne several children in earlier age, the advance in age did not materially add to the danger. The principal factors in the causation of insanity after delivery were want of proper nourishment, improper food, want of attention, and mental "shock," or anxiety occasioned by the loss of the child, the husband, or some near relative.

The Symptoms of Insanity.

We must next deal with the indications of insanity which should put nurses on their guard, such as extreme hysteria, nervousness, etc., in the past history of the patient. Indications were also furnished by the unreasoning caprices of patients, their likes and dislikes, their craving for food previously distasteful to them, and commonly of an indigestible character. This summary of the causes of mental distress in the puerperal state may, perhaps, lead my readers to think that insanity might occur in every case. Although, on an average, it did occur once in four hundred deliveries, yet classes of cases might be taken in which it was much less frequent; while, on the contrary, in women who had had insanity after their first delivery, probably more than one in four suffered after their second. A third of the cases occurred within the first five days, and fully another third before the end of the fourteenth day. Dr. Ripping stated that twenty-seven per cent. of his cases occurred between the fourth and seventh days, and this was, without doubt, the most critical period.

*(To be continued.)*

